# A new Palaearctic species of the subgenus *Lunatipula* (Diptera, Tipulidae) from the West Caucasus with a survey of the *caucasica* species group

**DOI:** 10.3897/zookeys.1048.67564

**Published:** 2021-07-13

**Authors:** Vladimir I. Lantsov, Valentin E. Pilipenko

**Affiliations:** 1 Tembotov Institute of Ecology of Mountain Territories of Russian Academy of Sciences, I. Armand str., 37a, Nalchik 360051, Russia Tembotov Institute of Ecology of Mountain Territories of Russian Academy of Sciences Nalchik Russia; 2 Lomonosov Moscow State University, GSP-1, Leninskie Gory, Moscow 119991, Russia Lomonosov Moscow State University Moscow Russia

**Keywords:** Crane flies, male and female terminalia, new species, Russia, *Tipula eleniya*

## Abstract

The *caucasica* species group in the subgenus Lunatipula is redefined and now consists of five species native to the Caucasus. *Tipula* (*L.*) *eleniya***sp. nov.** is described as new to science, and variations in the male terminalia in two populations are noted. Two subspecies (*quadridentataquadridentata* and *quadridentatapaupera*) are elevated to species rank. Detailed photo’s complement the descriptions of all five species (*caucasica*, *eleniya*, *paupera*, *quadridentata*, *talyshensis*), and data on ecology and distribution patterns are included as well as identification keys to males and females. *Tipula caucasica* is recorded from the West Caucasus and *Tipula quadridentata* is recorded from Dagestan (Russia) for the first time. Parallel evolution is traced in the male terminalia of the new species and in several non *caucasica* species group of Palaearctic *Lunatipula*.

## Introduction

There are 502 species in the subgenus Lunatipula Edwards in the world fauna (Oosterbroek 2021). This is the dominant taxon of crane flies in the Caucasus (Savchenko 1964, 1983), comprising at least 53 known species, which is ca. 32% of the total species of the family in this region (164). Riedel (1920) pioneered the work on crane flies of the Caucasus and neighboring regions, especially on the subgenus Lunatipula. He provided information on seven species of *Lunatipula*, three of which were described as new to science: Tipula (Lunatipula) caucasica Riedel, Tipula (Lunatipula) armata Riedel (synonym of *zaitzevi* Savchenko, 1952), and Tipula (Lunatipula) aurita Riedel. Savchenko (1954, 1957, 1964, 1968) made subsequent invaluable contributions to the knowledge of the regional fauna of the subgenus by describing 57 species, of which 32 were from the Caucasus and only two of these were subsequently synonymized (Oosterbroek 2021). The species group *caucasica* was first proposed by Savchenko (1964) to comprise four species endemic to the Caucasus. A number of subsequent studies have been published since on the diversity and ecology of some species of the subgenus Lunatipula from the Caucasus (Lantsov 1998, 2002, 2007, 2018). In this study we present new data on the taxonomic status, morphology, and distribution of the *caucasica* species group, provide a key to all currently known species, and describe *Tipula eleniya* Lantsov & Pilipenko as new to science. It was found in the Western Caucasus (Krasnodarskiy kray) at altitudes of 1200–2000 m. The new species is characterized by a projection on the inner gonostylus of the male, the structure of this shape is not known in any of the 502 known species, including the 360 Palaearctic species.

## Materials and methods

All crane flies were collected by sweep-net and then pinned. The genitalia were macerated in warm 10% KOH for ca. one hour to remove soft tissue, and then rinsed in distilled water. Cleared genitalia are preserved in glycerol in micro vials pinned with their respective specimens. Specimens were studied with an Olympus SZ61 stereo microscope. A Nikon d7000 digital camera equipped with combined Tamron 70–300 / 4–5.6 and EL-Nikkor 50/2.8 lenses or Mitutoyo M Plan Apo 10X Microscope objective lens was used to capture partially focused images of each specimen or structure. These were stacked using the Helicon Focus (version 7.6.4) software (http://www.heliconsoft.com/heliconsoft-products/helicon-focus). Photographs of lateral and dorsal view and details of the structure of head, pronotum and scutum were made with a Canon 5D Mark IV digital camera equipped with a Canon MP-E 65mm f/2.8 1–5 × macro lens and Canon Macro Twin-Lite MT-26EX-RT flash. Adobe Photoshop CC 2019 software was used to edit the pictures. Measurement were made with an MBS–10 microscope with a scale installed in the focal plane of the 8× eyepiece (divisions of scale: 0.1 mm at 8 × 1 magnification, 0.05 mm at 8 × 2 magnification, 0.025 at 8 × 4 magnification).

For citing label data on type specimens, a slash / separates each label. Square brackets [] are used to indicate additional information not on the original label. Original spelling is retained, including punctuation. In some cases, holotypes were marked by a red label without text and some paratypes were not initially marked. In such cases, specimens with locality labels corresponding to those in the publications of Savchenko (1964) or Savchenko and Kandybina (1987), were provided with a red label with the word “Holotype” or a white label with the word “Paratype,” and pinned with the specimen. These type specimens are deposited in the Zoological Institute of Russian Academy of Science, St. Petersburg, Russia. The holotype and paratypes of the Tipula (Lunatipula) caucasica were unavailable for this study.

We generally follow the terminology of Cumming and Wood (2017) except that for head morphology we follow Crampton (1943) and Savchenko (1983), and regarding wing venation, we follow Savchenko (1983). Veins are indicated by capitals; cells are indicated by lowercase letters.

All measurements of the new species were made on pinned material.

Species distributions are given according to Oosterbroek (2021).

The original descriptions and measurements by Riedel (1920) and Savchenko (1964) were taken into account in the redescriptions of the species.

Abbreviations for institutional and private collections used herein: **IEMT** Tembotov Institute of Ecology of Mountain Territories of Russian Academy of Sciences, Nalchik, Russia; **VPMC** private collection of Valentin E. Pilipenko, Moscow, Russia; **ZISP**Zoological Institute Russian Academy of Sciences, St. Petersburg, Russia. All specimens from the *caucasica* species group deposited in ZISP and collected before 1964 have been identified by E.N.Savchenko unless specifically noted.

## Taxonomy

### Family Tipulidae Latreille, 1802

#### Genus *Tipula* Linnaeus, 1758

##### Subgenus Lunatipula

Taxon classificationAnimaliaDipteraTipulidae

Edwards, 1931

52FEDC5D-FC63-5689-B524-1DC67EEADBF9

###### The *caucasica* species group.

**Diagnosis** (after Savchenko 1964 with additions). Medium sized grey species. Rostrum with nasus, wings with more or less distinct bluish tint in transmitted light; metakatepisternum with setae; abdomen slate or brownish grey with lateral intermittent dark brown stripe on tergites. Males with tergite 9 transverse with two or three concave notches at apex; inner gonostylus posteriorly simple; sternite 8 with paired appendages composed of short wide base with bristles and an elongate glabrous spine bent medially; no dense brush of setae between bases of paired appendages (except for *T. quadridentata*). Caucasian endemics. Savchenko included the following species in the *caucasica* group: *caucasica*, *quadridentataquadridentata*, *quadridentatapaupera*, *talyshensis*.

##### Tipula (Lunatipula) caucasica

Taxon classificationAnimaliaDipteraTipulidae

Riedel, 1920

219A9352-B860-5688-9DBE-B0947CC84DEA

**Figure 1.**
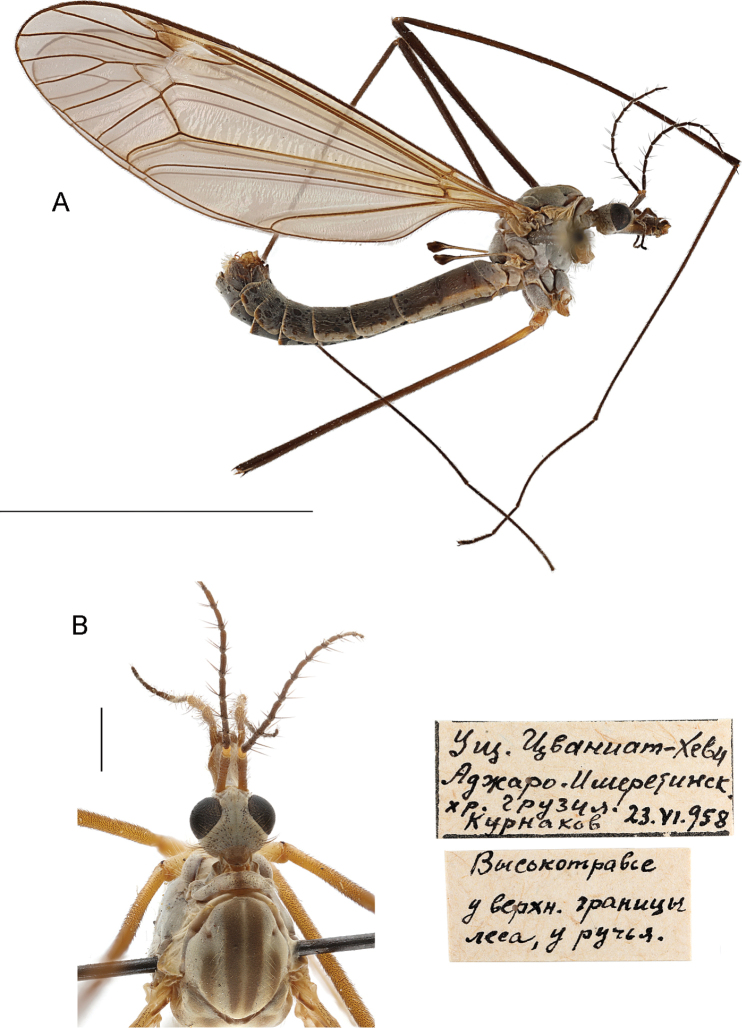
T. (Lunatipula) caucasica male habitus **A** general lateral view **B** head and thorax, dorsal view. Scale bars: 10 mm (**A**), 1 mm (**B**).

**Figure 2.**
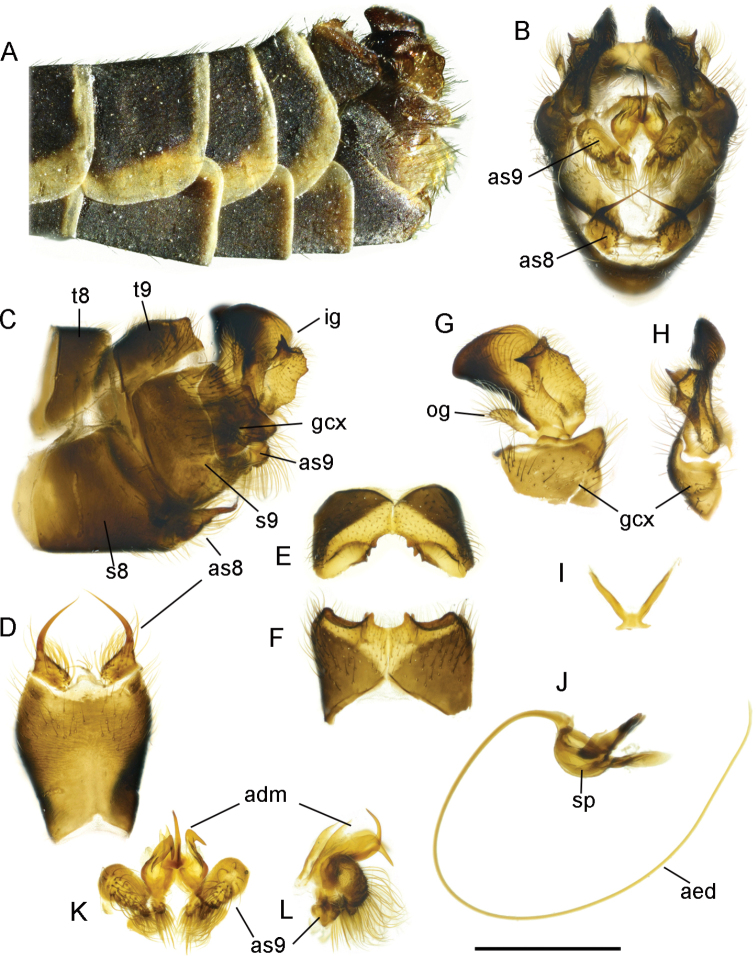
T. (Lunatipula) caucasica**A** terminal segments of male abdomen, lateral view (dry) **B–L** male terminalia (after KOH 10% treatment) **B** hypopygium, caudal view **C** hypopygium, lateral view **D** sternite 8, ventral view **E** tergite 9, caudal view **F** tergite 9, dorsal view **G** inner and outer gonostylus, lateral view **H** inner and outer gonostylus, caudal view **I** gonocoxal fragment, dorsal view **J** semen pump and aedeagus, lateral view **K** adminiculum and paired apical appendages of sternite 9, caudal view **L** adminiculum and paired apical appendages of sternite 9, lateral view. Abbreviations: adm–adminiculum; as 8–paired appendages of sternite 8; as 9–apical appendages of sternite 9; ig–inner gonostylus; gcx–gonocoxite; og–outer gonostylus; s8–sternite 8; s9–sternite 9; t9–tergite 9. Scale bar: 1 mm.

Figs 1 , 2 , 12A–C , 13A , 14A,F

Tipula (Lunatipula) caucasica Riedel 1920: 17 (type locality: Georgia, Mts’chet, prope Tiflis); Savchenko 1964: 390; Oosterbroek, Theowald 1992:104; Lantsov 2020: 294; Oosterbroek 2021.

###### Material examined.

Georgia • 1 male, 3 females; “ущ. Бани-Хеви бл. Боржоми, Аджаро-Имеретинск. хр. [Gorge Bani-Khevi near Borjomi, Adjaro-Imeretinsk. Ridge]; 26 Jun. 1958; Kurnakov leg.; ZISP • 1 male; “Цхра-Цхаро близ Бакуриани” [Tskhra-Tskharo near Bakuriani]; 29 Jun. 1958; Kurnakov leg.; ZISP •1 male; “Леберде, Менгрелия, Грузия” [Leberde, Mengrelia, Georgia]; 17 Jul. 1959; Savenko leg.; ZISP • 1 male; “Аджаро-Имеретинск. хр. Горы у Саирме, Грузия” [Adjaro-Imeretinsk. Ridge, mountains near Sairme, Georgia]; 23 May 1958; Kurnakov; ZISP • 2 males, 1 female; ”ущ. Цваниат-Хеви Аджаро-Имеретинск. хр. Грузия” [Tsvaniat-Khevi Gorge, Adjaro-Imeretinsk. Ridge, Georgia]; 23 Jun. 1958; Kurnakov leg; / “Высокотравье у верхн. границы леса у ручья” [High grass at the edge of the border of the forest by the stream]; ZISP • 2 males; “с. Отхара, Гудаутск. р-н, Абхазия, бер. речки, кусты.” [village Otkhara, Gudautsk. District, Abkhazia, river bank, bushes]; 30 Jun. 1958; Kurnakov; ZISP •2 males; “Абхазия, с. Отхара предгорья Бзыбского хр.” [Abkhazia, village Otkhara foothills of the Bzybsk Ridge]; 20 May 1958; Kurnakov; / “Дубово-грабовый лес, кусты Asalia” [Oak-hornbeam forest, bushes Asalia”]; ZISP; Azerbaijan • 1 male; “Белоканы, хр. Ахкемал. Азерб. 200 м” [Belokany, Akhkemal Ridge. Azerb.[aidjan] alt. 200 m]; 15 Jun. 1964; Pastukhov leg.; ZISP; Russia – **Krasnodarskiy kray** • 1 male; Sotshi prov. Pontica; 6 May 1932; B.Rohdendorf leg.; / “T.caucasica Ried det. Lacksch.” [terminalia in Canada balsam pinned with specimen]; ZISP • 1 female, Khosta, Caucasian Reserve, Tiso-samshitovaya rosha [Yew-and-Boxwood Tree Grove]; 43°31'656"N, 39°52'467"E; alt. 54 m; collected at light, 8 May 2018; V. Lantsov leg.; IEMT • 2 males, 2 females; same collection data as for preceding, 43°31'688"N, 39°52'561"E; alt. 35 m; 8 May 2018; V. Lantsov leg. / Lime-beech (*Tilia begoniifolia* and *Fagus orientalis*) forest with yew (*Taxus baccata*) and hornbeam (*Carpinus betulus*) in tree layer and butcher’s-broom (*Ruscus colchicus* – dominant and *Ruscus aculeatus*) in ground layer [one of the females was collected in the same community in a damp place near a yew log]; IEMT • 2 males, 2 females, same collection data as for preceding, 43°31'780"N, 39°52'518"E; alt. 98 m; 9 May 2018; V. Lantsov leg.; / Hornbeam – ash (*Carpinus orientalis*, *Fraxinus excelsior*) forest with addition of yew (*Taxus baccata*) and lime (*Tilia begoniifolia*) with blackberry (*Rubus sanctus*) and fig (*Ficus carica*) in shrub layer and with butcher’s-broom (*Ruscus colchicus*) in ground layer; ZISP • 4 males, 4 females, same collection data as for preceding, 43°31'775"N, 39°52'423"E; alt. 101 m; 9 May 2018; V. Lantsov leg.; / Hornbeam (*Carpinus orientalis*) forest with butcher’s-broom (*Ruscus colchicus*) and fern (*Asplenium scolopendrium*) in ground layer; ZISP • 1 male and 1 female (in copula), 1 female; same collection data as for preceding, 43°31'913"N, 39°52'463"E; alt. 153 m; 9 May 2018, V. Lantsov leg. / Ash-lime (*Fraxinus excelsior* + *Tilia begoniifolia*) forest with hornbeam (*Carpinus betulus*) and with black berry (*Rubus anatolicus*) and miscellaneous herbs in ground layer; IEMT • 2 males, 1 female, same collection data as for preceding, 43°31'825"N, 39°52'653"E; alt. 99 m; 10 May 2018, V. Lantsov leg.; IEMT • 1 male, same collection data as for preceding, 43°31'656"N, 39°52'467"E; alt. 54 m; V. Lantsov leg.; ZISP • 2 males, 1 female, same collection data as for preceding,43°32'257"N, 39°52'682"E; alt. 94 m; 10 May 2018, V. Lantsov leg.; / beech (*Fagus orientalis*) forest with addition of yew (*Taxus baccata*) and hornbeam (*Carpinus betulus*) with bunch grass (*Calamagrostis arundinacea*) in ground layer; IEMT • 6 males, 3 females; Krasnodar Territory, environs Shakhe River higher than Salokh-Aul; 5–8 Jun. 2011; V. Pilipenko leg.; VPMC; Russia – **Dagestan** • 1 female, “Хочал-Даг, альп. обл. Дагст. [Дагестан]” [Hochal-Dag, alp. Region Dagst. [Dagestan]]; 29 Jun. 1909 Mlokosevich leg.; ZISP • 1 female; Tlyaratinsky district, near village Salda, stream on the right bank of the river Dzhurmut; 41°58'388"N, 46°30'446"E; alt. 1792 m; 2 Jul. 2016, V. Lantsov leg.; / “Oak (*Quercus iberica*) sparse forest with *Populus tremula* and *Acer platanoides* in tree layer, *Rosa canina*, *Spiraea crenata* and *Cotoneaster integerrimus* in shrub, and clover (*Trifolium canescens*) and miscellaneous herbs in ground layer; ZISP • 2 males, 1 female; near village Betelda, stream on the left bank of the river Dzhurmut; 41°56'853"N, 46°32'424"E; alt. 1850 m; 3 Jul. 2016, V. Lantsov leg.; / Willow (*Salix caprea*) shrub with herbaceous layer (*Epilobium montanum*) (dominant), *Cardamine seidlitziana*, *Mentha caucasica*, *Veronica anagallis-aquatica* and *Myosoton aquaticum* community near spring above the flood plain of the left bank of the river; ZISP • 2 males; near village Genekolob, on the high left bank of the river Dzhurmut; 41°57'392"N, 46°31'276"E; alt. 2011 m; 4 Jul. 2016, V. Lantsov leg.; / Oak (*Quercus iberica*) forest with mountain-ash (*Sorbus aucuparia*), wayfaring tree (*Viburnum lantana*) in shrub layer [the lower border of a forest and a forb-cereal meadow]; ZISP • 2 males; same collection data as for preceding, 41°57'449"N, 46°31'228"E; alt. 1977 m; 4 Jul. 2016, V. Lantsov leg.; ZISP • 2 males, between the villages of Gerel and Genekolob, the right bank of the river Dzhurmut, 41°55'619"N, 46°34'468"E; alt. 1925 m; 5 Jul. 2016, V. Lantsov leg.; / Above the floodplain terrace with a rare herbaceous birch (*Betula raddeana*) forest with willow (*Salix caprea*) (coppice forest); ZISP.

###### Diagnosis.

Dark stripes medially on lighter background extending entire length of scutum. Inner gonostylus posteriorly with wedge-shaped projection directed anteriorly. Tergite 9 at apex with three rounded notches, middle one usually deepest and widest.

###### Redescription.

Adult male (Fig. 1A, B). General color grey. Body length 14–17 mm, wing 14–17.5 mm.

***Head.*** Light grey, sometimes bluish (Figs 1B, 13A). Rostrum whitish dorsally, flanks rusty or brownish with whitish bristles; nasus distinct, with long, whitish yellow setae; palpus grey. Frons with rows of brown setae along inner edge of eye. Vertex with faint dark median line, posteriorly with dark grey striation, with group of small rusty setae arranged fan-like on either side of glabrous medial area. Genae rusty; wide dark median stripe ventrally on rostrum, continuing along gula midline. Pharyngeal foramen framed with long, rusty hair-like setae. Postgenae light grey.

***Antenna*** (Fig. 1B) 13-segmented, if bent backwards just reaching wing base. Scape light brown or grey, depending on angle of rotation to light source, with slightly pronounced silvery pruinescence and brown horizontal striations. Pedicel yellow, sometimes brownish at base. Flagellomeres brownish, thickened at base, verticils subequal in length to corresponding flagellomeres.

***Thorax*** (Fig. 1B). Grey or blue-grey. Pronotum with dark grey median stripe. Katepisternum, anepisternum and meron grey, glabrous. Scutum with four longitudinal wide brownish stripes on lighter background; medial stripes extending length of scutum. Scutal lobes, scutellum and mediotergite grey with light bristles in dark brown sockets.

***Wings*** (Fig. 1A) greyish with a bluish tinge in transmitted light, stigma brown; narrow whitish area (lunule) extending to base of discal cell.

***Halteres*** (Fig. 1A) with light brown or dark-yellow stem, lighter yellow at base, covered with short small pale setae; knob dark brown to black.

***Legs*** with coxae light grey with long light hairs. Coxae may appear darker when specimen is rotated. Trochanters yellow. Femora yellow at base, rest of femora, tibiae, and tarsi dark brown.

***Abdomen*** (Figs 1A, 2A). Grey or dark grey; tergites with dark lateral stripe narrowly interrupted with whitish at posterior margins, lateral margins with wider whitish edge.

***Terminalia*** (Figs 2, 14A, F). Hypopygium (Fig. 2B, C) not much thicker than rest of abdomen, brown with moderately dense grey pruinescence, with golden yellow hairs. Gonocoxite (Fig. 2C, G) with broad tooth ventrally. Tergite 9 (Figs 2C, E, F; 14A) transverse, lateral corner broadly rounded, at apex with three rounded notches, middle one usually deepest and widest, bounded laterally by two large dentate projections with blunt apices, clearly visible when viewed from above (Figs 2F, 14A) (Riedel 1920; Savchenko 1964). When viewed from behind, three pairs of very short projections are visible (Fig. 2E). Outer gonostylus (Figs 2G, 14F) very small, wineglass shaped, elongated distally. Inner gonostylus posteriorly, with wedge-shaped projection (Figs 2G, 14F). Paired appendage of sternite 8 (Fig. 2D) with base ~ 2 × as long as wide, with thin rigid yellow setae along edge, not obscuring gap in middle; long, glabrous red-brown spine arising from base, bent, and intersecting medially. Paired appendage of sternite 9 (Fig. 2K, L) with thick pubescent apex. Gonocoxal fragment (Fig. 2I) flattened at the base. Semen pump and aedeagus as in Figure 2J.

**Female.** Body length 17–21.5 mm, wing 15.5–18 mm. Antennae with scape and pedicel brownish yellow. Legs stocky, black to brown, yellow at base, femora grey. Wings with noticeable bluish tint; whitish area (lunule) of wings barely reaching base of discal cell. Female terminalia (Fig. 12A–C) comparatively short. Tip of cercus upcurved, length of cercus ~ 0.8 mm. Hypogynial valve only slightly longer than width at base; length of hypogynial valve 0.2 mm. Sternite 9 and furca as in Figure 12A, C.

###### Comparison with closely related species.

This species differs from other species of the *caucasica* group primarily by the presence of a large, anteriorly directed sharp wedge-shaped projection located posteriorly on the inner gonostylus.

###### Elevation.

Adults were collected at altitudes ranging from sea level (Black Sea coast at 35 m) to 2011 m (in Dagestan).

###### Flight period.

From end of April to the beginning of July.

###### Habitat.

Specimens were captured in diverse forest habitats (see above) predominantly in mesophytic moderately moist deciduous or mixed communities with *Fagus orientalis*, *Taxus baccata*, *Carpinus orientalis*, *Quercus iberica*, *Populus tremula*, *Acer platanoides*, etc., sometimes in communities near springs with *Salix caprea*.

###### Distribution.

Endemic to the Caucasus. It was recently noted for Russia (Dagestan) for the first time (Lantsov 2020). Currently known from the West Caucasus (first record), East Caucasus and Transcaucasus.

###### Remarks.

According to Riedel (1920), a series of type specimens, four males, two females. “Mts’chet prope Tiflis [Georgia, Tbilisi], 6. 5. 1913 (Mus. Caucas. Ph. Zaitzev)” were deposited in the “Museum Caucasicum in Tiflis”. In the author’s publication, neither the holotype nor the paratypes are indicated. Presumably, they are not indicated on the material itself. Because this material turned out to be inaccessible to the authors of this article – it is not yet known whether this material has survived at all – there was no opportunity to designate lectotype and paralectotypes.

##### Tipula (Lunatipula) eleniya sp. nov.

Taxon classificationAnimaliaDipteraTipulidae

35B73B80-118E-5247-AD3A-67AD3A54266C

http://zoobank.org/2847C4F8-9727-4FC3-BECD-C552B75382C8

**Figure 3.**
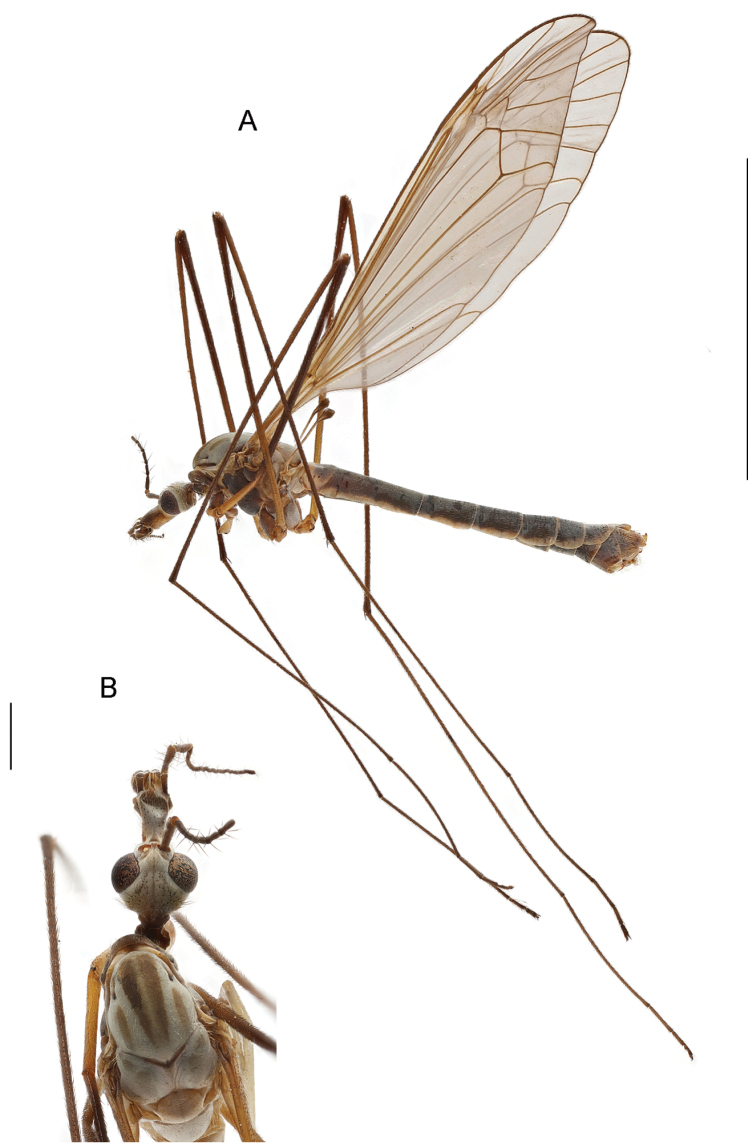
T. (Lunatipula) eleniya sp. nov., male habitus. Holotype **A** general lateral view **B** head and thorax, dorsal view. Scale bars: 10 mm (**A**), 1 mm (**B**).

**Figure 4.**
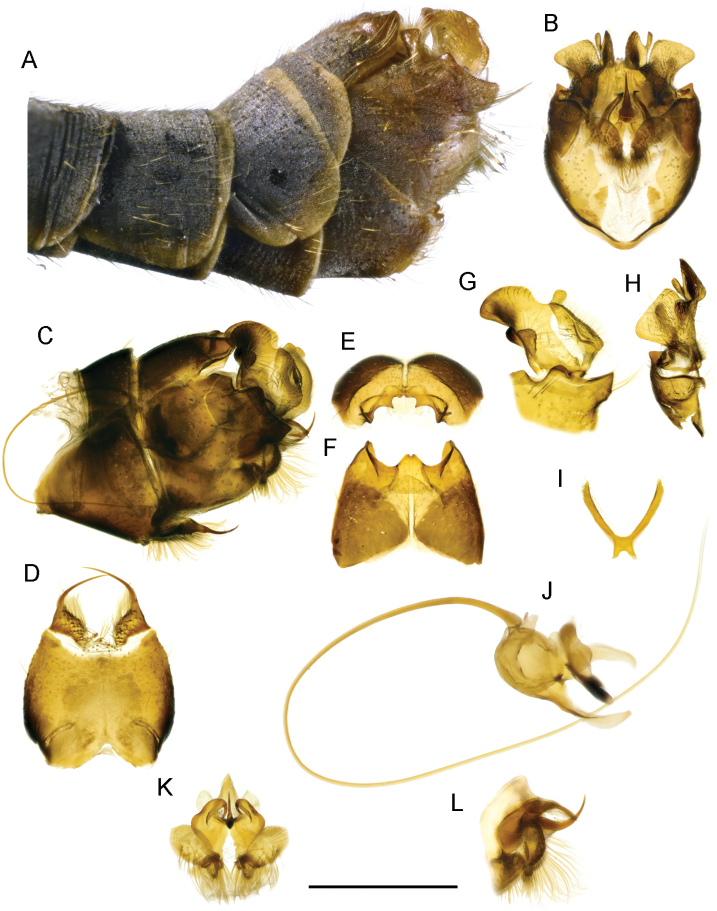
T. (Lunatipula) eleniya sp. nov. **A** terminal segments of male abdomen, lateral view (dry) **B–L** male terminalia (after KOH 10% treatment) **B** hypopygium (without sternite 8), caudal view **C** hypopygium, lateral view **D** sternite 8, ventral view **E** tergite 9, caudal view **F** tergite IX, dorsal view **G** inner and outer gonostylus, lateral view **H** inner and outer gonostylus, caudal view **I** gonocoxal fragment, dorsal view **J** semen pump and aedeagus, lateral view **K** adminiculum and paired apical appendages of sternite 9, caudal view **L** adminiculum and paired apical appendages of sternite 9, lateral view. Scale bar: 1 mm.

**Figure 5.**
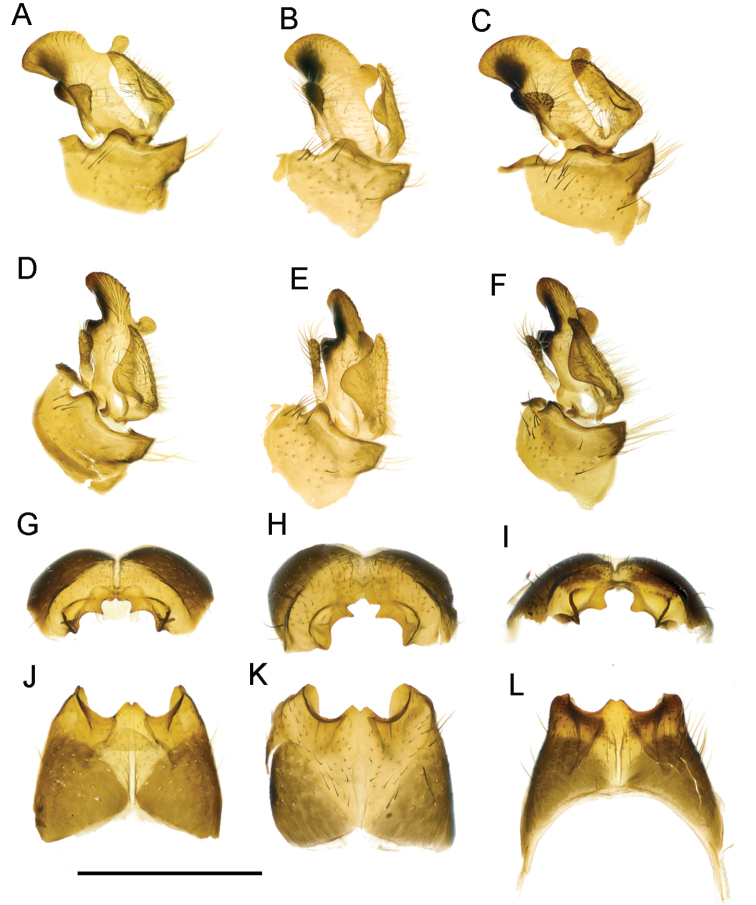
T. (Lunatipula) eleniya sp. nov., variation in the structure of the male terminalia **A, D, G, J** variant I (holotype) **B, E, H, K** variant II (paratype) **C, F, I, L** variant III (paratype) **A–C** inner and outer gonostylus, lateral view **D–F** inner and outer gonostylus, latero-caudal view **G–I** tergite 9, caudal view **J–L** tergite 9, dorsal view. Scale bar: 1 mm.

Figs 3 , 4 , 5 , 13B , 14B, G

###### Material examined.

***Holotype*:** Russia • 1 male; Krasnodarskiy Kray, Sochi env. Psekhako Mt., 43°41'28"N, 40°22'E; alt. ~ 2000 m; 14–18 Jun. 2008; K. Tomkovich leg.; ZISP. Holotype in good condition; however, left antenna, front right and left hind legs missing. ***Paratypes***: Russia • 1 male; same data as for holotype • 2 males; Krasnodarskiy Kray, Kamyshanova Polyana env. [Biological station of Krasnodar State University], 44°16'91"N, 40°04'46"E; alt. 1200 m; 26 Jul. 2018; V..Pilipenko leg.; ZISP.

###### Diagnosis.

Male. General coloration grey with silver pruinescence. Tergite 9 with deep rounded notches either side of slightly grooved cone-shaped projection. Sternite 8 with a pair of small appendages, each bearing a long medially curved spine, broad base of appendages with fringe of long whitish hairs. Outer gonostylus small, triangular, slightly thickened distally, covered with long setae. Inner gonostylus with small rod-like outgrowth in middle of outer edge.

###### General description.

Adult (Fig. 3A). Male body length 15.7 mm, wing length 16.2 mm, haltere 2.5 mm, nasus 0.13 mm, rostrum 0.8 mm, scape 0.6 mm, pedicel 0.15 mm, 1^st^ flagellomere 0.5 mm. Length (mm) of leg segments, fore (1), mid (2), and hind (3); successively femur, tibia, 1^st^ and 2^nd^ tarsomeres: 1 (9.7; 10.4; 8.5; 3.8), 2 (9.8; 10.5; 9.0; 4.5) and 3 (10.0; 13.0; 9.0; 4.5). Length (mm) of 3^rd^, 4^th^, and 5^th^ tarsomeres of all three pairs of legs, approximately the same: 1.0; 0.6; 0.5 mm, respectively.

###### Description.

***Head*** (Figs 3, 13B) grey with silvery pubescence. Rostrum grey dorsally, ventrally yellow-brown with sparse light rusty setae in black sockets. Nasus distinct, appearing triangular in dorsal aspect, covered with long, sparse procumbent setae. Frons and gula bare. Vertex (Fig. 13B) framed by rows of sparse setae alongside eye, glabrous in center with indistinct narrow longitudinal dark grey line. Genae, including lateral part of rostrum, with very sparse fine pale bristles. Tempora and postgenae with sparse black setae.

***Antennae***, bent backwards, reaches base of abdomen. Scape grey with silvery pruinescence, pedicel dirty yellow brown. Flagellomeres dark brown with verticils on slightly thickened bases, longest verticils subequal to length of respective flagellomere.

***Thorax*** (Fig. 3A, B) grey with silvery pruinescence, and short sparse whitish hairs. Pronotum dark brown with a weak dark stripe medially, and short sparse whitish bristles. Scutum (Fig. 3B) with four brownish grey longitudinal stripes, lateral stripe shorter, medial stripes narrowly separated by dirty yellow area. Katepisternum, anepisternum, katepimeron, anepimeron and meron grey, bare, with silvery pruinescence; scutellum and mediotergite light grey with silvery pruinescence; scutal lobes with wide dark grey stripe, bordered by sparse whitish setae and with long yellow setae anterolaterally at wing base. Scutellum framed anteriorly with brown, with sparse yellow setae laterally, and with vague narrow dark grey medial line. Mediotergite light grey with sparse yellow setae and with silvery pruinescence. Laterotergite grey with silvery pruinescence and sparse erect short brown setae.

***Wings*** (Fig. 3A) with typical venation for the subgenus, transparent with brownish tin, (viewed in transmitted light). Stigma dirty yellow. Oblique lunule proximal to stigma. Setae on costal and subcostal veins; group of short black macrotrichia distally on *R* and base of *R_1_*. Distal section of *R_s_*, *R_4 + 5_*, *r-m*, base of pentagonal discal cell lighter and weakened. Stem of cell *m_1_* approximately half length of cell. Apex of vein *Cu*_1_ sharply curved at wing margin.

***Halteres*.** Stem grey with base covered with short white setae, knob dark grey.

***Legs*.** Coxae light grey with silvery pruinescence; laterally with long sparse whitish yellow setae; medially with very short yellowish setae. Trochanters yellow with long yellow setae. Femora brown, narrowly yellow at base, covered with adpressed short brown setae; tibiae brownish; tarsal segments dark brown with short, adpressed brown setae. Tibial spur formula 1-2-2. Tarsal claws with a small tubercle at the base (magnification 10 × 4.5); with short but clearly visible arolium between ones.

***Abdomen*** (Figs 3A, 4A). Tergites and sternites grey with silvery pruinescence, and sparse fine short golden setae. Tergites with lateral dark grey intermittent stripe, no distinct dorsal stripe. Tergites laterally and distally edged with whitish-yellow, proximal segments with thinner distal edge, becoming wider on caudal segments.

***Terminalia*** (Figs 4, 5, 14B, G). Hypopygium grey to dark grey, moderately thickened, with silvery pruinescence (Fig. 4A–C). Tergite 9 with two deep rounded notches flanking a median cone-shaped projection, the apex of which has a small groove (Figs 4E, F; 5G–L, 14B). The dorsolateral edges of the tergite 9 convex, curved inward. Sternite 8 (Fig. 4D) with a pair of small appendages bearing long medially curved spine, base of appendages with fringe of long whitish hairs not obscuring gap between. Appendages of sternite 9 (Fig. 4K, L) abundantly pubescent. Outer gonostylus (Figs 4G, 5A–F, 14G) small, triangular, slightly thickened distally, covered with long bristles. Inner gonostylus (Figs 4G, H; 5A–F, 14G) with small rod-shaped outgrowth in middle of outer edge (Figs 4G, 14G), twice as long as wide at base, slightly narrower medially, tip obliquely truncate. Posterior part of inner gonostylus a wide triangular plate with short sparse brown bristles in inner surface, long whitish setae in outer one. Gonocoxite (Figs 4G, 5A–C) with two wide dentate outgrowths, ventral one longer, pointed, dorsal one rounded; long yellowish setae laterally on gonocoxite. Gonocoxal fragment (Fig. 4I) with short forked extension at base. Semen pump and aedeagus as in Figure 4J.

**Female** unknown.

###### Variation.

Three variants exist of the small rod-like outgrowth of the inner gonostylus: variant I (holotype – Sochi env., Psekhako Mt, alt. 2000 m) (Fig. 5A, D), variant II (paratype) (Fig. 5B, E) and variant III (paratype) (Fig. 5C, F). (variants II and III from Kamyshanova polyna env., alt. 1200 m). There are also some differences in the shape of tergite 9 (Fig. 5G–L). Given the paucity of material, and the minor differences observed, the authors consider this to be intraspecific variability.

###### Comparisons to similar species.

The new species is readily separable from all other species of the subgenus by the presence of an outgrowth medially, on the outer edge of the inner gonostylus (Figs 4G; 5A–F; 14G).

###### Elevation.

Adults were collected at altitudes ranging from 1200–2000 m.

###### Flight period.

Adults are active from middle of June through the end of July.

###### Habitat.

Mixed moderately moist mesophytic plants, shady communities that include common hornbeam (*Carpinus betulus*), oriental beech (*Fāgus orientālis*), Nordman fir (*Abies nordmanniana*), ash vulgaris (*Fraxinus excelsior*), field maple (*Acer campestre*), colchis holly (*Ilex colchica*), etc.

###### Distribution.

Endemic to the Caucasus: currently known from the West Caucasus.

###### Etymology.

Tipula (Lunatipula) eleniya sp. nov. is named after the mother of the first author, Elena Nikolaevna Lantsova.

##### Tipula (Lunatipula) paupera

Taxon classificationAnimaliaDipteraTipulidae

Savchenko, 1964

0273951C-175D-51F0-98B0-12BAF0F9EB34

**Figure 6.**
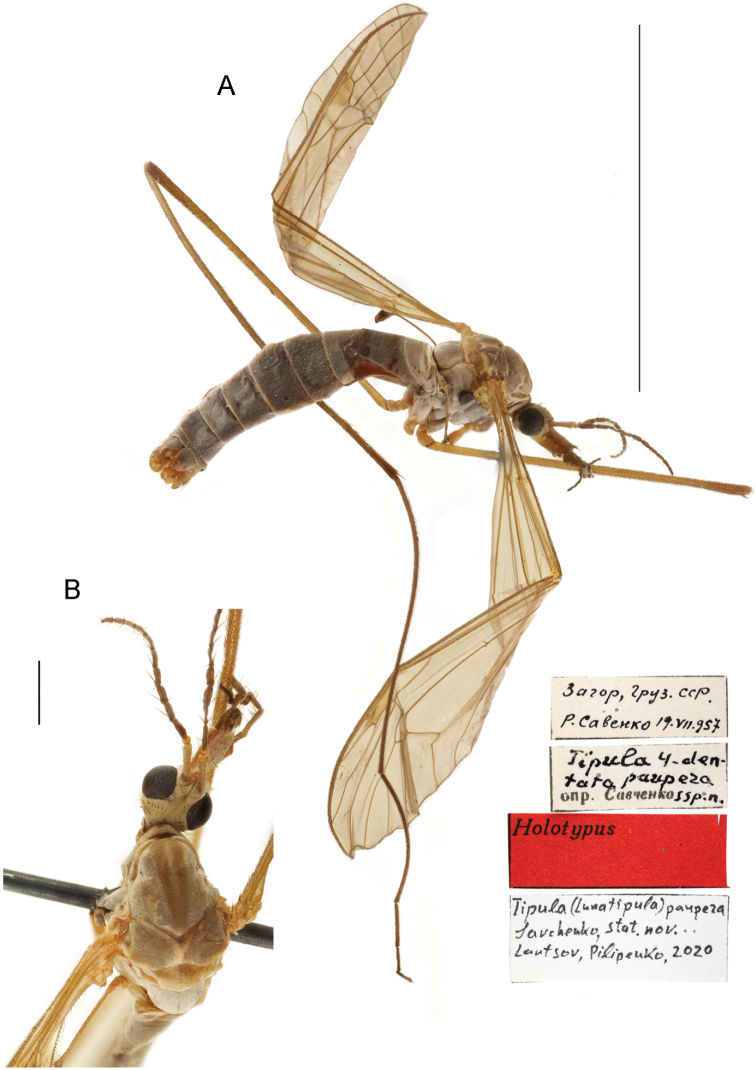
T. (Lunatipula) paupera, male habitus **A** general lateral view **B** head and thorax, dorsal view. Scale bars: 10 mm (**A**), 1 mm (**B**).

**Figure 7.**
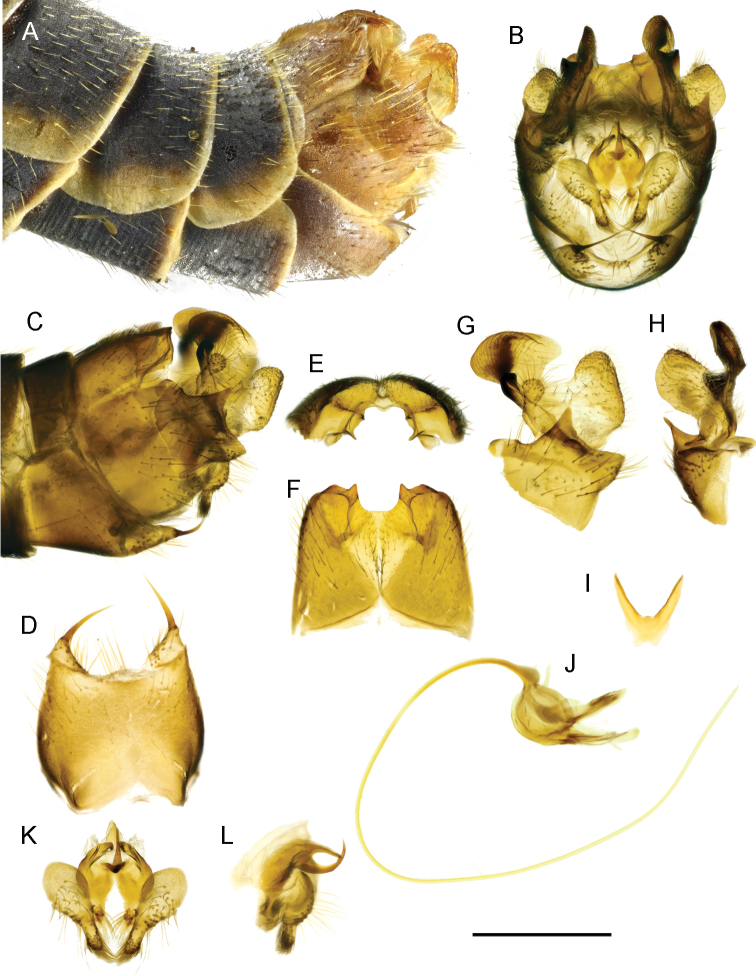
T. (Lunatipula) paupera**A** terminal segments of male abdomen, lateral view (dry) **B–L** male terminalia (after KOH 10% treatment) **B** hypopygium, caudal view **C** hypopygium, lateral view **D** sternite 8, ventral view **E** tergite 9, caudal view **F** tergite 9, dorsal view **G** inner and outer gonostylus, lateral view **H** inner and outer gonostylus, caudal view **I** gonocoxal fragment, dorsal view **J** semen pump and aedeagus, lateral view **K** adminiculum and paired apical appendages of sternite 9, caudal view **L** adminiculum and paired apical appendages of sternite 9, lateral view. Scale bar: 1 mm.

Figs 6 , 7 , 13C , 14C, I

Tipula (Lunatipula) quadridentata paupera Savchenko 1964: 393 (type locality: Georgia, Zagor Pass, Svanetiya); Oosterbroek, Theowald 1992: 118; Oosterbroek 2021.

###### Material examined.

***Holotype*:** Georgia •1 male; “Загор [Загар Сванетия], Груз. ССР” [Zagor Pass Svanetiya, Gruz. SSR. Georgian Soviet Socialist Republic]; alt. 2623 m; 19 Jul. 1957; R. Savenko leg.; ZISP. / “*Tipula 4-dentata paupera* ssp. n. det. Savchenko” / “Holotypus” [Holotype not initially marked; red label] / “Tipula (Lunatipula) paupera Sav., stat. nov. Lantsov, Pilipenko, 2020” [white label]. Thorax (prescutum, scutum, scutellum) smeared, obscuring coloration. Preservation of legs: forelegs both with only trochanters; midlegs: left with part of femur, right only with femur; hind legs: right missing. Wings slightly crumpled. ***Paratypes*.** Georgia •1 male; “ур. Лаедиль (Корулдаш [Корулдаши] – Загар)” [Laedil [territory] (Koruldash [Koruldashi] – Zagar)]; 5 Aug. 1957; Savenko leg.; ZISP. / “Paratypus” [Paratype not initially marked; red label] / “Tipula (Lunatipula) paupera Sav., stat. nov. Lantsov, Pilipenko, 2020” [white label] • 2 males; “Спуск с перевала Басса в Накру [в долину р. Накра], Ставропольский кр.” [Descent from the Bassa Pass to Nakra [to the valley of the river Nakra], Stavropol kr. [Stavropol Territory – in error, Georgia, Svanetiya]; 5 Aug. 1956; L. Arens leg.; ZISP. / “Paratypus” [Paratype not initially marked; red label] / “Tipula (Lunatipula) paupera Sav., stat. nov. Lantsov, Pilipenko, 2020” [white label].

###### Diagnosis.

Male. Gonocoxite with two elongate, pointed teeth, one dorsally and one ventrally. Tergite 9 with two projections posteriorly, separated by deep wide notch. Paired appendages of sternite 8 widely spaced, base shorter than wide, gap between not masked by setae. Apical appendages of sternite 9 elongate, narrow distally, with dense bundle of relatively short golden yellow setae at tip.

###### Redescription.

Adult male (Fig. 6A, B). General color light grey. Body length 15.5 mm, wings 16.5 mm.

***Head*** (Figs 6B, 13C). Rostrum dorsally light grey to whitish with silvery pruinescence, dark brown procumbent setae, base of rostrum dorsally light beige, laterally dirty rusty, yellow. Nasus well defined with longer whitish procumbent setae. Palpus dark brown. Vertex (Fig. 13C) light sandy color with soft silvery pruinescence, dark grey thin median line extending to occiput; nine or ten short brown setae, behind antenna, four or five longer brown setae alongside eye. Row of long brown setae on temporal region and around occipital foramen. Gula and gena (including rostrum ventrally) yellowish-sandy to light brown, with medially enfolded region ventrally marked by moderately broad, light brown median line; gena near eyes light yellow; postgena yellow to sandy.

***Antennae*.** Scape light grey with silvery pruinescence, pedicel yellow with subtle brown ring at base. Flagellomeres brown.

***Thorax*** (Fig. 6A, B). Pronotum grey with dark broad median stripe with blurred edges. Scutum with four brown stripes, central pair more pronounced, widener anteriorly with dirty yellow between. Scutal lobes brown with silvery pruinescence. Scutellum and mediotergite with thin fuzzy grey median line; scutellum yellow to light grey; mediotergite light grey with silvery pruinescence and scattered whitish short bristles. Katepisternum, anepisternum, katepimeron, anepimeron and meron light grey with silvery pruinescence, glabrous.

***Wings*.** Transparent, without noticeable marble pattern, with light brown stigma. Longitudinal veins with macrotrichia.

***Halteres*.** Stem light brown to dirty yellow, knob brown.

***Legs*.** Coxae light grey with silvery pruinescence and long, whitish bristles; trochanters light brown; femora yellow at base, light brown with darkened tips, with adpressed dark brown bristles.

***Abdomen*** (Figs 6A, 7A). Grey with short whitish bristles. Posterior and lateral margins of tergites with thin whitish edging.

***Terminalia*** (Figs 7, 14C, I). Hypopygium not thickened. Tergite 9 and gonocoxites with silvery pruinescence. Gonocoxite (Figs 7G, 14I) with two teeth. Tergite 9 (Figs 7E, F; 14C) distally with projection on either side of deep, wide central notch. Paired appendages of sternite 8 (Fig. 7D) widely separated, basal section shorter than width, gap between not masked by setae. Appendage of sternite 9 (Fig. 7K, L) with dense bundle of relatively short golden-yellow setae at tip. Gonocoxal fragment as in Figure 7I, semen pump and aedeagus as in Figure 7J.

**Female.** Unknown.

###### Status.

The species was described and treated as a subspecies of *T. quadridentata*. It is elevated here to species rank because of the presence of a number of differences of the species from *T. quadridentata* and all other species of the *caucasica* species group (see below).

###### Comparison with closely related species.

This species differs from all other species of the *caucasica* group by the number and arrangement of setae dorsally on the head (Fig. 13C), the deep median notch at the apex of tergite 9 (Figs 7F, 14C), and the more widely spaced appendages of sternite 8 (Fig. 7D). It differs from *T. eleniya* sp. nov., *T. quadridentata*, and *T. caucasica* in the shape and presence of the dense bundle of short setae on the appendages of sternite 9 (Fig. 7K). This species is similar to *T. quadridentata* in having two teeth on the gonocoxite (Figs 7G, 14I, J).

###### Elevation.

The holotype was probably collected at 2623 m (height of Zagar Pass) and one of the paratypes was collected “on the descent from the Bassa Pass”, the height of which is 3057 m. It can be assumed that this species occurs in high-mountainous habitats.

###### Flight period.

Adults were collected from the last third of July to early August.

###### Habitat.

Data absent.

###### Distribution.

Endemic to the Caucasus-currently known from the southern slopes of the Greater Caucasus (Georgia).

##### Tipula (Lunatipula) quadridentata

Taxon classificationAnimaliaDipteraTipulidae

Savchenko, 1964

E104034B-F2F2-51DB-8C14-5337C9D914B4

**Figure 8.**
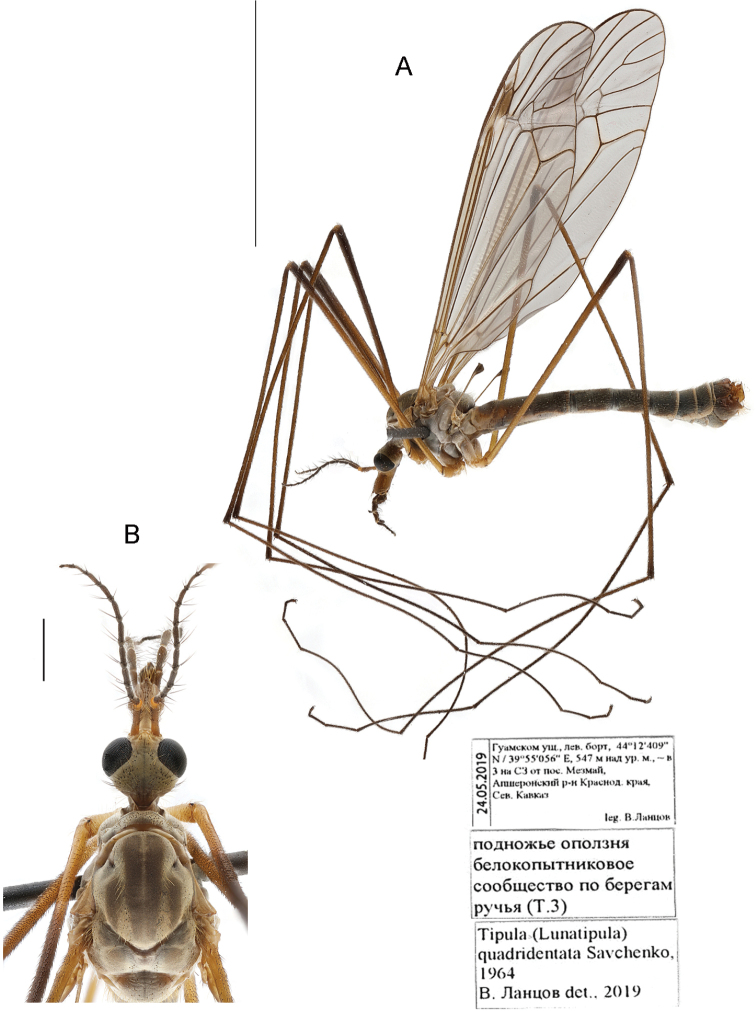
T. (Lunatipula) quadridentata, male habitus **A** general lateral view **B** head and thorax, dorsal view. Scale bars: 10 mm (**A**), 1 mm (**B**).

**Figure 9.**
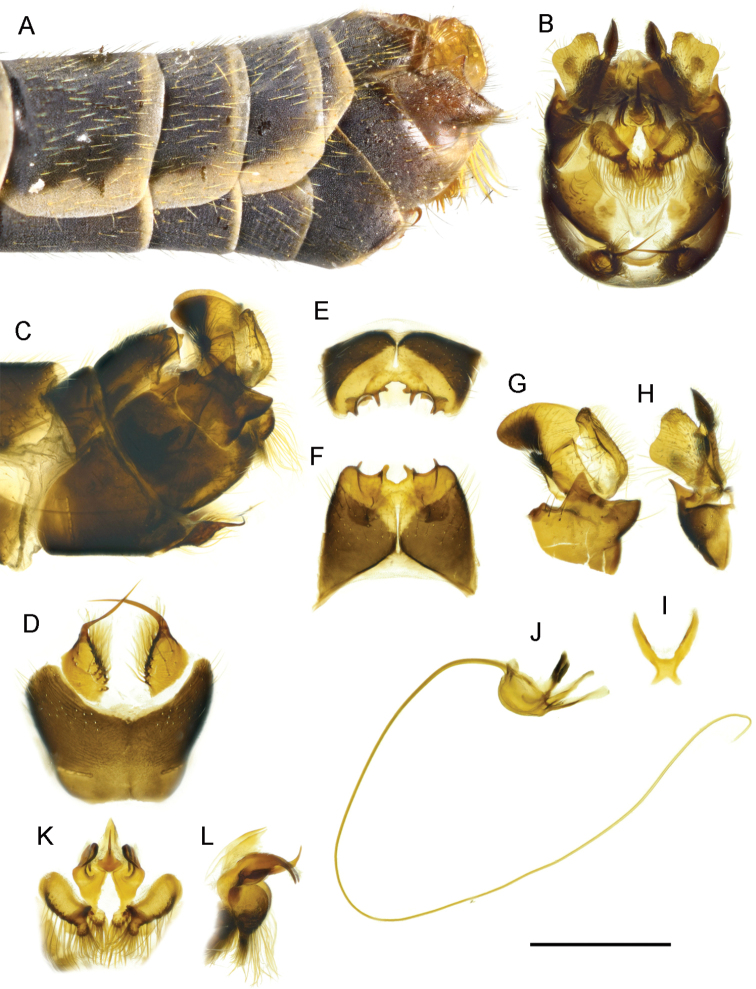
T. (Lunatipula) quadridentata**A** terminal segments of male abdomen, lateral view (dry) **B–L** male terminalia (after KOH 10% treatment) **B** hypopygium, caudal view **C** hypopygium, lateral view **D** sternite 8, ventral view **E** tergite 9, caudal view **F** tergite 9, dorsal view **G** inner and outer gonostylus, lateral view **H** inner and outer gonostylus, caudal view **I** gonocoxal fragment, dorsal view **J** semen pump and aedeagus, lateral view **K** adminiculum and paired apical appendages of sternite 9, caudal view **L** adminiculum and paired apical appendages of sternite 9, lateral view. Scale bar: 1 mm.

Figs 8 , 9 , 12D–F , 13D , 14D,J

Tipula (Lunatipula) quadridentata quadridentata Savchenko 1964: 392 (type locality: Russia, near Stavropol); Oosterbroek and Theowald 1992: 118; Oosterbroek 2021.

###### Material examined.

***Holotype*.** Russia • 1 male; ”околицы Ставрополя, байрачн.[ый] лес” [near Stavropol, bairak forest] [small forest in steppe ravines]; 25 May 1954; [S.] Medvedev leg.; ZISP. / “южн. скл. под пологом [леса]” [southern slopes under the canopy forest] / “T. (Lunatipula) quadridenta sp. n., опр. Е.Савченко” [det. E. Savchenko]. The specimen is very badly damaged and glued together. The head is glued to the thorax; the abdomen is broken in half and glued together; two legs of uncertain position are glued to the specimen, one without coxa and trochanter, and the other missing the last four tarsal segments. **Paratypes.** Russia •1 male; “Старый лес к югу от Ставрополя” [Old forest south of Stavropol]; 25 May 1954;[S.] Medvedev leg.; ZISP. / “ниж. часть сев. склона” [lower part of northern slope] • 1 female, “Георгиевское лесничество Туапсинск. р-н” [Georgievskoe forestry Tuapse District]; 21 May 1954; K. Arnoldi leg.; ZISP •1 male, same collection data as for preceding; 22 May 1954 • 3 males, 2 females; “г. Лысая, 800–900 м, Туапсинск. р-н” [mount Lysaya, alt. 800–900 m, Tuapse District]; 26 May 1954; K. Arnoldi leg.; ZISP; / “вершинная луговина и опушка леса 800–900 m” [summit meadow and forest edge, alt. 800–900 m].

###### Additional material.

Russia • 9 males, 8 females; “курорт “Горячий Ключ” хр. Котх.[ский] Краснодар.[ский] кр. дуб.[овый] лес” [Goryachiy Klyuch resort Koth[skiy] Ridge, Krasnodar.[sky] District oak. Forest]; 18 May 1956; Gilyarov leg.; ZISP / “T. (Lunatipula) quadridentata sp. n. опр. Е.Н.Савченко” • 1 female, “м. [мыс] Пенай, к югу от Новороссийска” [m. (cape) Penay, south of Novorossiysk] 24. V. [1]956; Gilyarov leg.; ZISP • 1 male, 1 female; “окр. ст. Смоленской, Сиверского р-на, Краснодар. кр.” [near Smolensk station, Siversky Region, Krasnodar District] 20 May 1963; Savchenko leg.; / “опуш.[ка] смеш.[анного] предгорн.[ого] леса” [edge of mixed foothill forest]; ZISP • 1 male; “окр. ст. Кривенковской, Туапс.[инского] р-на Краснодар.[ского] края” [around village Krivenkovskaya, Tuapse Region of Krasnodar District]; 25 May 1963; Savchenko leg.; / “опушка листв.[енного] леса у реки, вдоль горн.[ого] потока” [the edge of the foliage forest near the river, along the mountain stream]; ZISP • 1 male; Khosta, Krasnodarskiy Kray, Caucasian Reserve, Tiso-samshitovaya rosha [Yew-and-Boxwood Tree Grove]; 43°32'014"N, 39°52'621"E; alt. 135 m; 10 May 2018; V. Lantsov leg.; / Fern-butcher community on the rocky slopes of the right side of the gorge of the Khosta River; IEMT • 1 male; Khosta, Krasnodarskiy Kray, Caucasian Reserve, Tiso-samshitovaya rosha [Yew-and-Boxwood Tree Grove]; 43°31'656"N, 39°52'467’’ E; alt. 54 m; 12 May 2018, V. Lantsov leg.; / collected on light; IEMT • 2 males (in alcohol); North Caucasus, Krasnodarskiy Kray, near village Mezmay; 44°11'291"N, 39°58'090"E; alt. 808 m; 15 May 2018; V. Lantsov leg.; / Beech (*Fagus orientalis*), fruit tree (*Pyrus* sp. and *Malus* sp.) forest with *Fraxinus excelsior* in under growth, *Sambucus nigra*, *Cornus mas*, *Rosa canina*, *Crataegus* sp., in shrub layer and sedge *Carex pendula*, cereals and herbs in ground layer [a leveled area of the light part of the forest, possibly a site of a wild pear-apple orchard 50–70 years old]; IEMT • 1 male; Krasnodar Territory, Apsheron District, environs of village Mezmay, Guam Gorge, left slope, 44°12'409"N, 39°55'056"E, alt. 547 m; 24 May 2019; V. Lantsov leg.; / Landslide foot, community with butterbur [*Petasites albus*] as dominant along the banks of the stream, open places at the edge of the forest; ZISP • 8 males (in alcohol); Krasnodar Territory, Seversky District, in vicinity of village Thamaha, 3 May 2016; S. Kustov leg.; ZISP • 7 males, 1 female (in alcohol) ; Krasnodar Territory, Seversky District, environs of village Plancheskaya, 3 May 2016. S. Kustov leg.; ZISP • 5 males (in alcohol); Krasnodar Territory, environs of village Bolshoy Utrish; 1 May 2008; E. Hachikov. leg.; ZISP • 3 males (in alcohol); Krasnodar Territory, municipality Anapa, environs of village Sukko, Kvashin’s Gorge, Dolgaya Niva territory; 44°47'20"N, 37°28'33"E; alt. 67 m; 6–8 May 2016; S. Kustov, V. Gladun. leg.; ZISP • 5 males (in alcohol) ; Krasnodar Territory, river Shakhe Gorge; 43°52'46"N, 39°50'00"E; 2 May 2012; V. Pilipenko leg.; VPMC • 3 males; (in alcohol); Khosta, Krasnodarskiy Kray, Caucasian Reserve, Tiso-samshitovaya rosha [Yew-and-Boxwood Tree Grove]; 43°32'014"N, 39°52'621"E; 8 May 2012; V. Pilipenko leg.; VPMC • 4 males (in alcohol); Krasnodar Territory, 13 km to the N from Sochi, Sukhoy Canyon; 43°32’N, 39°56’E; 5 May 2014; V. Pilipenko leg.; VPMC; RUSSIA – Dagestan • 5 males; Makhachkala, Tarki Distr.; 42°56'57"N, 47°29'41"E; alt. 220 m; 1 May 2019., V. Pilipenko leg.; cemetery on the hillside; VPMC • 1 male, 1 female; same locality; 10 May 2019., V. Pilipenko leg.; VPMC • 1 male, Tarki-Tau Mt.; 42°56'28"N, 47°28'08"E; alt. 450 m; 2 May 2019; V. Pilipenko leg.; oak forest; VPMC.

###### Diagnosis.

Tergite 9 with four widely spaced teeth and with three rounded notches distally; caudal margin of gonocoxite with two dentate projections. Paired appendages of sternite 8 with wide base bearing thick yellow setae distally covering gap between. Cercus long and straight; hypogynial valve only slightly longer than width at base.

###### Redescription.

Adult male (Fig. 8A, B). General body coloration grey. Body length 15–16 mm, wings 15.5–17 mm.

***Head*** (Figs 8B, 13D). Vertex dirty grey with longitudinal thin dark grey midline. Rostrum grey dorsally with long brown setae mixed with shorter white setae, but glabrous at base, laterally and ventrally dirty yellow (nearly rusty). Nasus well developed, with long, whitish procumbent setae. Frons light grey and yellow with group of 2–4 small brown setae behind each antenna . Gula grey, glabrous with narrow brown stripe. Gena light grey near eyes.

***Antennae*.** Framing around antennal sockets yellow. Scape light brown, pedicel yellow, flagellomeres brown; verticils not longer than corresponding flagellomeres.

***Thorax*** (Fig. 8A, B). Dark grey with silvery pruinescence. Pronotum dark grey; katepisternum dorsally with groups of whitish setae. Scutum with 4 brownish stripes (Fig. 8B), spaces between stripes grey with long whitish bristles. Mediotergite grey with whitish bristles.

***Wings*** (Fig. 8A). Transparent, without noticeable marble pattern, with light brown pterostigma. Longitudinal veins with macrotrichia.

***Halteres*.** Stem light brown to yellowish, knob brown.

***Legs*.** Femora light brown with procumbent dark brown bristles. Tarsal claws without noticeable tooth at base.

***Abdomen*** (Figs 8A, 9A). Dark grey with short whitish bristles. Tergites with distal and lateral whitish edging.

***Terminalia*** (Figs 9, 14D, J). Hypopygium not thickened. Tergite 9 and gonocoxite with silver pruinescence. Upper margin of gonocoxite with two dentate projections. Tergite 9 (Figs 9E, F; 14D) at apex with four widely spaced teeth and with three rounded notches, narrower middle notch bounded by small flat projection bearing sharp thorn beneath; small backwards projecting spine beneath tergite 9. Outer and inner gonostyli (Figs 9G, H; 14J) without significant features. Paired appendages of sternite 8 (Fig. 9D) large, wide base with fringe of thick yellow setae hiding gap between (apparent on pinned specimens). Paired apical appendages of sternite 9 as in Figure 9K, L. Gonocoxal fragment (Fig. 9I) with characteristic small V-shaped fork at base. Semen pump and aedeagus as in Figure 9J.

**Female.** Body length 17.5–19.5 mm, wings 16–18.5 mm. Scape and pedicel brownish yellow. Terminalia (Fig. 12D–F). Cercus long and straight. Hypogynial valve (Fig. 12E) only slightly longer than wide at base. Sternite 9 and furca as in Figure 12D, F.

###### Comparison with closely related species.

This species differs from other species of the *caucasica* species group (in the male) by tergite 9 with four widely spaced teeth and with three small notches at the apex and a small spine beneath (Figs 9E, F; 14D). Paired appendages of sternite 8 is broadest, with thick yellow bristles that cover the gap between; in the other species this gap is not obscured. *Tipula quadridentata* is similar to *T. paupera* by having two teeth on the edge of the gonocoxite and similar coloring of the antenna. The outer gonostylus is somewhat larger than in *T. caucasica*. The gonocoxal fragment is similar to that of *T. talyshensis* (Figs 9I, 11I). The female has a parallel-sided, straight cercus whereas it is upturned at the tip in *T. caucasica* and broader at base in *T. talyshensis*.

###### Elevation.

Adults were collected at altitudes ranging from sea level (54 m in Tiso-samshitovaya rosha) to 800–900 m (in Mount Lysaya, Tuapse district).

###### Flight period.

Adults were collected from throughout the month of May.

###### Habitat.

Specimens are found in moderately humid woody deciduous communities.

###### Distribution.

Endemic to the Caucasus; currently known from the West Caucasus (northern and southern slopes; Krasnodar and Stavropol Territory) and from the East Caucasus (Dagestan; first record).

##### Tipula (Lunatipula) talyshensis

Taxon classificationAnimaliaDipteraTipulidae

Savchenko, 1964

1D18358B-8391-55CF-B3A1-33D56252432A

**Figure 10.**
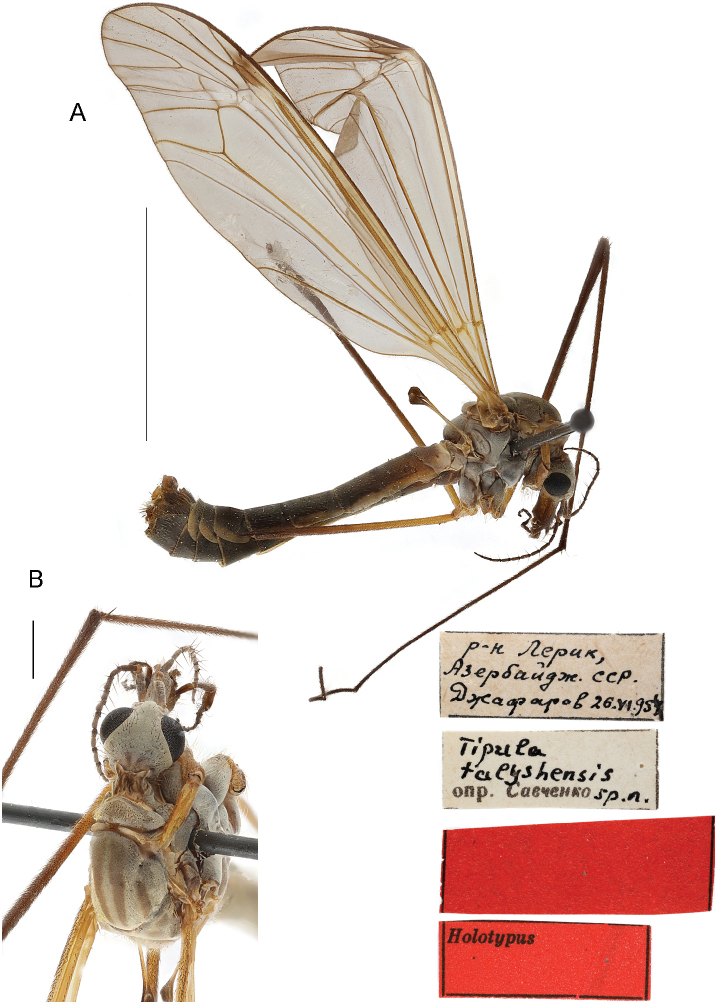
T. (Lunatipula) talyshensis, male habitus **A** general lateral view **B** head and thorax, dorsal view. Scale bars: 10 mm (**A**), 1 mm (**B**).

**Figure 11.**
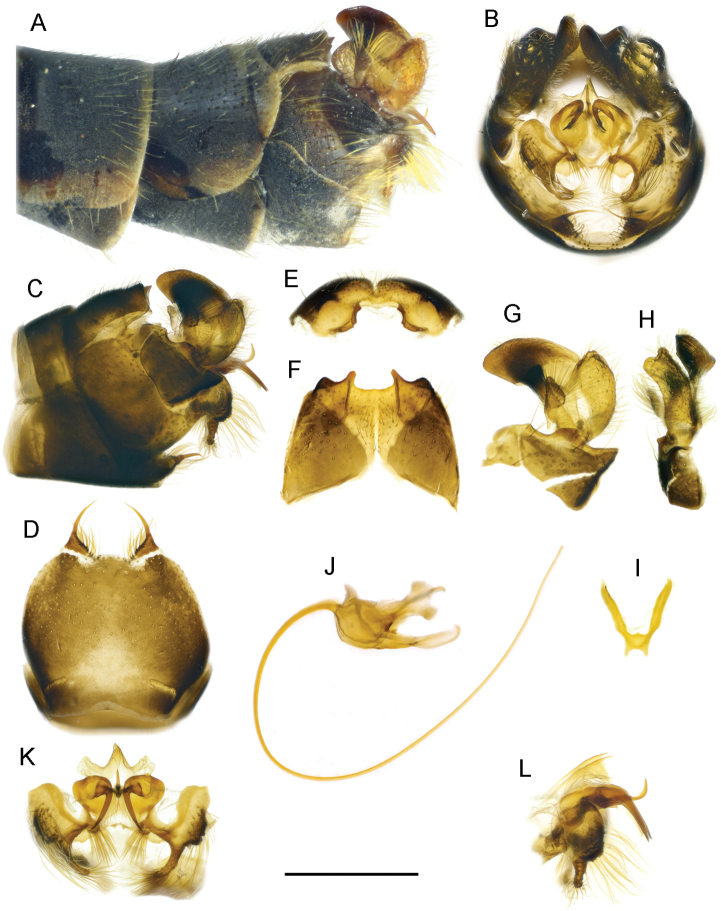
T. (Lunatipula) talyshensis**A** terminal segments of male abdomen, lateral view (dry) **B–L** male terminalia (after KOH 10% treatment) **B** hypopygium, caudal view **C** hypopygium, lateral view **D** sternite 8, ventral view **E** tergite 9, caudal view **F** tergite 9, dorsal view **G** inner and outer gonostylus, lateral view **H** inner and outer gonostylus, caudal view **I** gonocoxal fragment, dorsal view **J** semen pump and aedeagus, lateral view **K** adminiculum and paired apical appendages of sternite 9, caudal view **L** adminiculum and paired apical appendages of sternite 9, lateral view. Scale bar: 1 mm.

**Figure 12.**
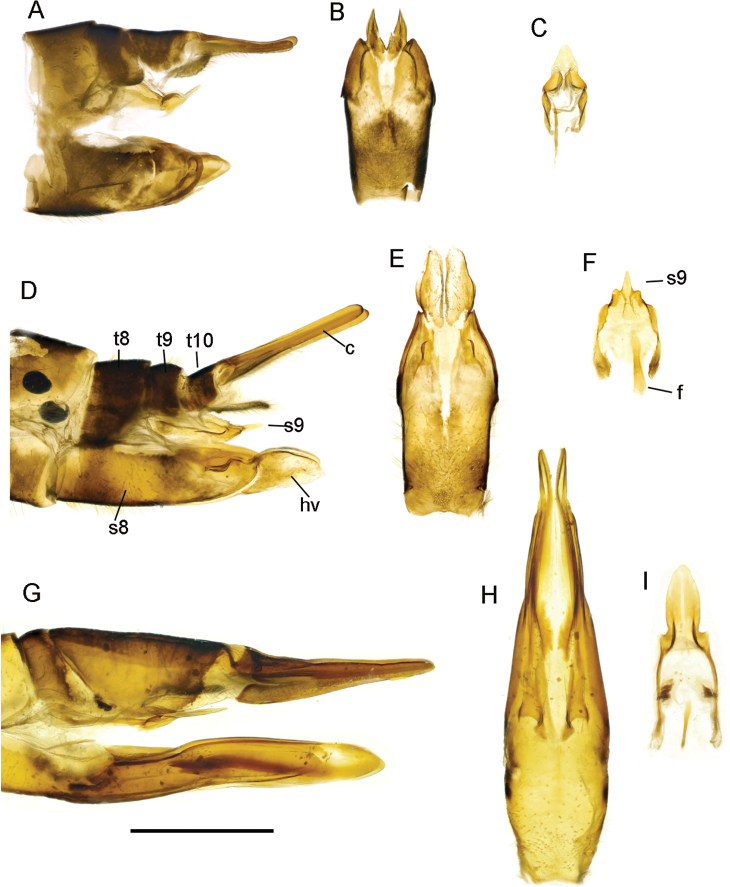
Female terminalia of *caucasica* species group **A–C**T. (Lunatipula) caucasica**D–F**T. (Lunatipula) quadridentata**G–I**T. (Lunatipula) talyshensis**A, D, G** ovipositor, lateral view **B, E, H** ovipositor ventral view **C, F, I** sternite 9 and furca, lateral view. Abbreviations: c–cerci; f–furca; hv–hypogynial valve; s–sternite; t–tergite. Scale bar: 1 mm.

**Figure 13.**
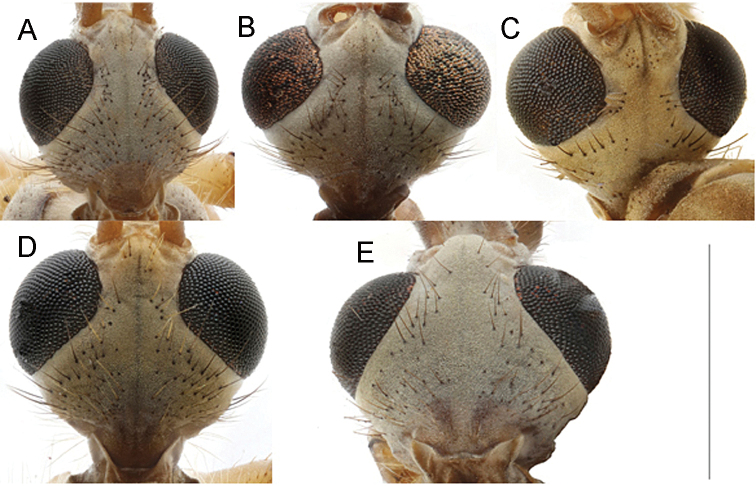
Heads of *caucasica* species group, dorsal view **A**T. (Lunatipula) caucasica**B**T. (Lunatipula) eleniya sp. nov. **C**T. (Lunatipula) paupera**D**T. (Lunatipula) quadridentata**E***T.* (*Lunatipula*) *talyshensis*. Scale bar: 1 mm.

**Figure 14.**
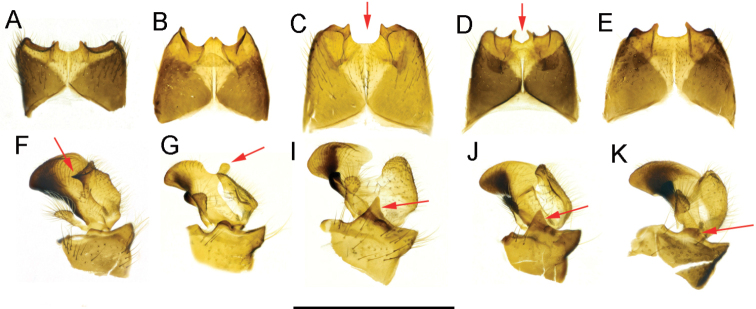
Comparisons of some components of male terminalia of the *caucasica* species group **A, F**T. (Lunatipula) caucasica**B, G**T. (Lunatipula) eleniya sp. nov. **C, I**T. (Lunatipula) paupera**D, J**T. (Lunatipula) quadridentata**E, K**T. (Lunatipula) talyshensis**A–E** tergite 9, dorsal view **F–K** inner and outer gonostylus, lateral view. Arrows point to central notch at apex of tergite 9 (**C, D**), to the wedge-shaped projection posteriorly on inner gonostylus (**F**), to the rod-like outgrowth behind anterior beak of inner gonostylus (**G**), to the anterior (dorsal) margin of gonocoxite (**I, J, K**). Scale bar: 1 mm.

Figs 10 , 11 , 12G–I ; 13E , 14E, K

Tipula (Lunatipula) talyshensis Savchenko 1964: 391 (type locality: Azerbaijan, Lerik region); Oosterbroek, Theowald 1992: 121; Oosterbroek 2021.

###### Material examined.

***Holotype*:** Azerbaijan •1 male, “р-н Лерик, Азербайдж. ССР 26. VI. [1]954 Джафаров” [Lerik region, Azerbaijan. SSR. [Azerbaijanskaya Soviet Socialist Republic] [alt. ~1115 m, 38°46'31"N, 48°24'55"E]; 26 Jun. 1954; Jafarov; / “*Tipula talyshensis* det. Savchenko sp. n.” [white label] / [Original red label without text] / “Holotypus” [red label]; ZISP. Holotype in good condition (Fig. 10A, B), however, some legs missing: fore legs – left missing, right up to femur; mid legs – left present, right missing; hind legs – left and right up to femur. ***Paratypes*.** Azerbaijan •2 males, 2 females; same data as for holotype [printed on white paper] / “Paratypus” [Paratype not initially marked; red label]; ZISP.

###### Diagnosis.

Male. Tergite 9 at apex with a denticle on either side of wide, shallow, flat median notch. Paired apical appendages of sternite 9 narrower distally, bearing tassel of long golden setae. Female. Hypogynial valve several times longer than width at base.

###### Redescription.

Adult male (Fig. 10A, B). General body coloration grey. Body length 15–20 mm, wings 18–19.5 mm.

***Head*** (Fig. 13E). Vertex light grey with indistinct median grey line, glabrous in middle and around eye with rows of setae between. Rostrum dorsally dark grey, whitish at base, ventrally light brown with grey median stripe. Nasus with long, whitish procumbent setae. Tempora and genae light grey to whitish (color depending on angle of inclination) with faint silvery pruinescence.

***Antennae*.** Scape dark grey with rusty bristles, pedicel yellowish with indistinct brown line in middle, flagellum brown.

***Thorax*** (Fig. 10A, B). Pronotum grey with whitish setae. Scutum (Fig. 10B) with four brownish stripes on grey, short whitish setae between central and side stripes. Scutal lobe grey with faint silvery pruinescence, anterolaterally with group of whitish setae. Pleura and coxae grey with silvery pruinescence; katepisternum dorsally with sparse whitish setae, anepisternum and meron glabrous. Scutellum and mediotergite grey with whitish setae, scutellum with dark grey median line visible as specimen is rotated.

***Wings*** (Fig. 10A). Translucent, grey, with light brown pterostigma. Longitudinal veins *C*, *Sc*, and *R* and bases of *A_1_* and *A_2_* with macrotrichia.

***Halteres*.** Stem yellowish with light setae, knob brown.

***Legs*.** Coxae grey with silvery pruinescence and long, whitish setae. Femora light brown with procumbent dark brown setae. Claws of fifth tarsal segment without spine at base.

***Abdomen*** (Figs 10A, 11A). Dark grey with short whitish setae. Posterior margins of tergites without noticeable light edging, lateral margins with wide whitish to yellowish edging.

***Terminalia*** (Figs 11, 14E, K). Hypopygium (Fig. 11A, B, C) not thickened. Tergite 9 (Figs 11E, F; 14E) trapezoidal, apex with rather wide, shallow median notch bounded by a denticles. Gonocoxites (Figs 11G, 14K) with large ventral tooth, dorsal tooth truncated at apex. Outer gonostylus without significant features (Fig. 11G). Inner gonostylus as in Figures 11G, H; 14K. Paired appendages of sternite 8 (Fig. 11D) relatively small, with fringe of hairs along inner edge. Apical appendages of sternite 9 (Fig. 11K, L) narrowed distally, bearing tassel of long golden setae. Gonocoxal fragment, semen pump and aedeagus as in Figure 11I, J, respectively.

**Female.** Adult female body length 18–25 mm, wings 18–18.5 mm. Similar to male. Cercal hypogynial valve (Fig. 12G, H) several times longer than wide at base. Sternite 9 and furca as in Figure 12I.

###### Comparison with closely related species.

This species differs from other species of the *caucasica* group by the broad shallow notch at the apex of tergite 9, the tassel of golden setae at the tip of the apical appendage of sternite 9, and by the small size of the paired appendages of sternite 8. The unusually long hypogynial valve of the female distinguishes this species from that of *T. caucasica* and *T. quadridentata*, the other known females of the *caucasica* group.

###### Elevation.

Adults were collected at altitudes ~ 1115 m.

###### Flight period.

Adults were collected on 26 June.

###### Habitat.

No data.

###### Distribution.

Endemic to the Caucasus – currently only known from Talysh (Azerbaijan).

### Key to males and females of the *caucasica* species group

**Males**

**Table:** 

1	Inner gonostylus with rod-like outgrowth behind anterior beak (Fig. 14G)	***T. eleniya* sp. nov.**
–	Without such an outgrowth behind anterior beak on the inner gonostylus (Fig. 14F, I–K)	**2**
2	Wedge-shaped projection posteriorly on inner gonostylus (Fig. 14F)	***T. caucasica* Riedel, 1920**
–	Without such a wedge-shaped projection posteriorly on inner gonostylus	**3**
3	Anterior (dorsal) margin of gonocoxite with sharp dentate projection in addition to smaller more ventral tooth (Fig. 14 I, J). Adminiculum and paired apical appendage of sternite 9 as in Figure 7K or 9K	**4**
–	Anterior (dorsal) margin of gonocoxite with only traces of a projection at most (Fig. 14K). Apical appendage of sternite 9 narrowed distally, bearing tassel of long golden setae (Fig. 11K, L)	***T. talyshensis* Savchenko, 1964**
4	Tergite 9 at apex with four projections, central notch between middle projections small and shallow (dorsal view) (Fig. 14D). Paired appendage of sternite 8 with thick yellow setae on base covering space between. Setal arrangement on vertex as in Figure 13 D. Adminiculum and paired apical appendages of sternite 9 as in Figure 9K	***T. quadridentata* Savchenko, 1964**
–	Tergite 9 at apex with only two projections, central notch between quite wide and deep (dorsal view) (Fig. 14C). Gap between paired appendage of sternite 8 usually exposed, not masked by setae. Arrangement of setae on vertex as in Figure 13C. Adminiculum and paired apical appendage of sternite 9 as in Figure 7K	***T. paupera* Savchenko, 1964**

**Females (known for three species of the *caucasica* group only)**

**Table:** 

1	Hypogynial valve long, more than three times longer than width at base (Fig. 12G, H)	***T. talyshensis* Savchenko, 1964**
–	Hypogynial valve short, length approximately equal to width at base (Fig. 12A, B, D, E)	**2**
2	Cercus upcurved distally (Fig. 12A) ***T. caucasica* Riedel, 1920**	
–	Cercus straight (Fig. 12D)	***T. quadridentata* Savchenko, 1964**

## Discussion

All species belonging to the *caucasica* species group are morphologically quite distinct including *T. quadridentata* and *T. paupera*, previously considered as subspecies. Their status as separate species is beyond doubt: each is distinguishable by the structure of tergite 9 as well as additional distinctive features mentioned in the descriptions and comparisons above, as well as in the keys. For *T. caucasica*, this is a characteristic wedge-shaped projection posteriorly on the inner gonostylus (Fig. 14F); for *T. eleniya*, it is a unique rod-shaped outgrowth of the inner gonostylus (Fig. 14G); *T. talyshensis* differs by the unique structure of the paired appendage of sternite 9 (Fig. 11K). The inner gonostyli of *T. quadridentata*, *T. paupera*, and *T. talyshensis* are similar in lateral view (Fig. 14I, J, K), but these species differ in size, shape, and coverage of the paired appendages of sternite 8 (Figs 7D, 9D, 11D). *T. eleniya*, *T. quadridentata*, and *T. talyshensis* are similar in the structure of the gonocoxal fragment, semen pump, and aedeagus (Figs 4I, J; 9I, J; 11I, J). The color and arrangement of the setae on the vertex of the head in *T. paupera* distinguishes it from all other species of the group (Fig. 13C). The variability seen in the outgrowth of the inner gonostylus of *T. eleniya* (Fig. 5) probably indicates the plasticity of this species and confirms the need for additional studies.

Females of only three of the five species of the *caucasica* species group are known, and they differ sufficiently in the structures of the cerci, hypogynial valves, and sternite 9 (Fig. 12), as reflected in the identification key. Of interest would be the immature stages of the species of the *caucasica* species group, about which nothing is yet known.

Zoogeographically, the *caucasica* species group belongs to the Caucasian subgroup, a part of the eastern Mediterranean group, which is, in turn, a part of the Mediterranean species complex (Savchenko 1983). These species are narrow-range endemics to the Caucasus. Ecologically, they are confined to mesophytic habitats within the forest belt, occurring in a fairly wide range of altitudes and can be classified as species characteristic of deciduous forest and mixed communities.

### Parallel morphology in the subgenus Lunatipula in the structure of internal gonostylus in Palaearctic species

The inner gonostylus of T. (L.) eleniya was compared with those of the 502 known species including the 360 Palaearctic species of the subgenus Lunatipula. No direct matches were found, but outgrowths of various shapes on the middle of the inner gonostylus were found in some Palaearctic species: two species from Turkey (Tipula (Lunatipula) auriculata Mannheims, 1963 and Tipula (Lunatipula) horsti Theischinger, 1982), one from China (Tipula (Lunatipula) oreada Alexander, 1933), and two from Kyrgystan (Tipula (Lunatipula) milkoi Pilipenko, 2005 and Tipula (Lunatipula) zarnigor Savchenko, 1954). The latter species has also been recorded in Tajikistan and northeastern Afghanistan (Oosterbroek 2021). The species belong to the same subgenus, but are classified in different species groups and are geographically dispersed in this regard, it can be assumed that these various outgrowths to be independently derived and therefor examples of parallel evolution.

## Supplementary Material

XML Treatment for Subgenus Lunatipula

XML Treatment for Tipula (Lunatipula) caucasica

XML Treatment for Tipula (Lunatipula) eleniya

XML Treatment for Tipula (Lunatipula) paupera

XML Treatment for Tipula (Lunatipula) quadridentata

XML Treatment for Tipula (Lunatipula) talyshensis
